# Malignant Transformation and Long-Term Outcome of Oral and Laryngeal Leukoplakia

**DOI:** 10.3390/jcm12134255

**Published:** 2023-06-25

**Authors:** Botond Bukovszky, János Fodor, Erika Tóth, Zsuzsa S. Kocsis, Ferenc Oberna, Örs Ferenczi, Csaba Polgár

**Affiliations:** 1 Department of Oncology, Semmelweis University, 1122 Budapest, Hungary; 2 Department of Oral Diagnostics, Faculty of Dentistry, Semmelweis University, 1088 Budapest, Hungary; 3 Center of Radiotherapy and the National Tumor Biology Laboratory, 1122 Budapest, Hungary; 4 Department of Surgical and Molecular Pathology and the National Tumor Biology Laboratory, 1122 Budapest, Hungary; 5 Multidisciplinary Centre of Head and Neck Tumours and the National Tumor Biology Laboratory, 1122 Budapest, Hungary

**Keywords:** oral and laryngeal leukoplakia, risk of malignant transformation, survival with cancer

## Abstract

Background: Oral or laryngeal leukoplakia has an increased risk for malignant transformation but the risk of the two anatomical sites has not been compared to each other yet. Materials and Methods: Clinical data of 253 patients with leukoplakia (oral = 221 or laryngeal = 32) enrolled from January 1996 to January 2022 were analyzed. One hundred and seventy underwent biopsy and 83 did not. The mean follow-up time was 148.8 months. Risk factors for the malignant transformation of leukoplakia were identified using Cox proportional hazard models. Results: In the oral or laryngeal group, the rate of cancer was 21.7% and 50% (*p* = 0.002), respectively. The 10-year estimated malignant transformation was 15.1% and 42% (*p* < 0.0001), respectively. The laryngeal group had an increased risk of malignant transformation (*p* < 0.0001). The 5-year estimated survival with leukoplakia-associated cancer for the oral or laryngeal group was 40.9% and 61.1% (*p* = 0.337), respectively. Independent predictors of malignant transformation in the oral group were dysplasia and the grade of dysplasia of the leukoplakia, and in the laryngeal group, dysplasia had a significant impact. The malignant transformation rate was low for oral patients without biopsy or with no dysplasia, 3.9% and 5.1%, respectively. The malignant transformation occurred over 10 years. Conclusions: Patients with dysplastic leukoplakia have an increased risk of malignant transformation, but the risk is higher with laryngeal than with oral leukoplakia. There is no significant difference between the groups regarding survival with leukoplakia-associated cancer. Oral patients with no dysplastic lesions have a low risk of malignant transformation. A complete excision and long-term follow up are suggested for high-risk patients to diagnose cancer in an early stage and to control late (over 10 years) malignant events.

## 1. Introduction

Leukoplakia is the most common potentially malignant disorder of head and neck cancer. The range of malignant transformation from leukoplakia to squamous cell carcinoma amounts from 0.13% to 34% for oral leukoplakia and from 0% to 64.7% for laryngeal leukoplakia [1,2,3]. This large variation in the malignant transformation rates is in part caused by the criteria applied for a leukoplakia diagnosis, a geographical location, differences in study populations, possible etiological factors and length of follow up [3]. A prediction of the malignant potential is traditionally based on the histologically determined severity of the dysplasia. An increase of the grade of dysplasia increases the risk of malignant transformation into squamous cell carcinoma [2,3,4]. Moderate/severe dysplasia bears a much higher risk of cancer evolution than mild dysplasia [5,6]. A clinical examination does not accurately predict the risk of malignancy. More than half of the reported leukoplakia lesions with biopsies showed no dysplasia [5,7]. Since 2007, leukoplakia has been limited only to a clinical diagnosis defined by the exclusion of other white lesions such as oral lichen planus, white sponge nevus, nicotine stomatitis, leukoedema, etc. [8]. To date, therapy strategies in the management of leukoplakia focus on “watch-full waiting”, a non-surgical application of local therapeutics and photodynamic therapy, serial incision biopsies or excision biopsy and laser vaporization depending on the determined grade of dysplasia and clinically apparent extension of the leukoplakia [9,10,11]. The aim of this study was to examine the association between the risk of malignant transformation and dysplasia in oral or laryngeal leukoplakia and to highlight the importance of the time course of the disease and follow-up strategies for these patients.

## 2. Materials and Methods

A chart review of all patients treated at the National Institute of Oncology Budapest, Hungary with laryngeal or oral leukoplakia over a 16-year period (January 1996 to January 2022) was performed. In the computerized institutional database, the number of patients with oral or laryngeal leukoplakia was 221 and 32, respectively. The histopathology of leukoplakia from the biopsy material was classified as no dysplasia, dysplasia and carcinoma. Dysplasia was classified as mild, moderate and severe (grade I–II–III). The pathology department in our institution uses the World Health Organization (WHO) classification for oral and laryngeal dysplasia [12,13]. Initial management consisted of biopsy, surgical excision, CO_2_ laser vaporization or observation only. Re-biopsy was performed if there was any deterioration in symptoms (such as the voice quality, swallowing or pain) or appearance of the lesion (endoscopically for laryngeal and to the naked eye in the case of oropharyngeal lesions). The histological findings were classified as follows: no dysplasia, dysplasia (grade I–II–III) or cancer. The treatment consisted of surgery with the removal of the lesion, CO_2_ laser treatment or observation. First, and before any treatment, we always took at least one biopsy before laser vaporization. In addition, surgical excisions were performed for dysplastic leukoplakia with a histological examination of the entire lesion. Regular follow up (two visits/year) was suggested for patients.

To assess the associations between the different study variables and the risk of developing cancer, a survival analysis was performed using the Kaplan–Meier [14] method. The number of patients progressing towards malignancy was evaluated in each of the groups. The follow-up period was defined as the interval from the time of a leukoplakia clinical diagnosis to death or the last follow up. The following survival endpoints were used: death from leukoplakia-associated cancer for cancer-specific survival, and the time to an appearance of cancer for malignant transformation-free survival. The survival curves were compared with a log-rank test. The effect of the possible prognostic factors on the probability of the incidence of leukoplakia-related cancer was examined in a Cox regression model [15]. Statistical differences in proportions and means were assessed with a Fisher-exact test and chi-squared test. All tests were two-sided, and *p* values of ≤0.05 were accepted for statistical significance. GraphPad Prism (GraphPad Prism version 5.01 for Windows, GraphPad Software, San Diego, CA, USA) and IBM SPSS Statistics for Windows (version 25.0, Armonk, NY, USA: IBM Corp) program packages were used for a data analysis.

## 3. Results

The mean or median follow-up time was 148.8 months and 144 months (range: 14–328 months), respectively. Seventeen patients presented with in situ or invasive cancer, and half of the invasive cancers had III–IV stages. In 47 patients, the cancer was developed during the follow-up time, between 6 and 204 months (mean time: 53.6 months). Six patients developed cancer after 120 months. The early-stage cancer (stage 0–I–II) rate was 48.4% (31 of 64). In total, 11 of the 64 cancer patients (in situ cancers are also included) are currently alive, 40 died of leukoplakia-associated head and neck cancer, 3 died of second primary cancer and 10 died of internal diseases. The average survival time with cancer was 64.3 months (10–221 months). The 5-year estimated survival with leukoplakia-associated cancer for patients with oral or laryngeal leukoplakia was 40.9% and 61.1% (*p* > 0.337), respectively. The number of biopsy/patients (prior to malignant transformation) was 1/148, 2/17, 3/4 and 4/1. Dysplasia progressed in 20 of 22 patients with multiple biopsies (oral, 13; laryngeal, 7). The average time to the malignant transformation of laryngeal leukoplakia or oral leukoplakia patients was 55.6 months (range: 6–204 months) and 52.7 months (range: 6–204 months), respectively (*p* = 0.913). The grade of dysplasia had a significant effect on the time to malignant transformation with oral leukoplakia. The mean metastasis transformation-free survival with a low grade or with a high grade was 88.0 and 11.3 months, respectively (*p* < 0.0001). In the laryngeal group, the difference between the two grades was not significant (*p* = 0.982). The crude rate of malignant events and the 10-year estimated malignant transformation rate using characteristics are shown in Table 1. 

**Figure 1:**
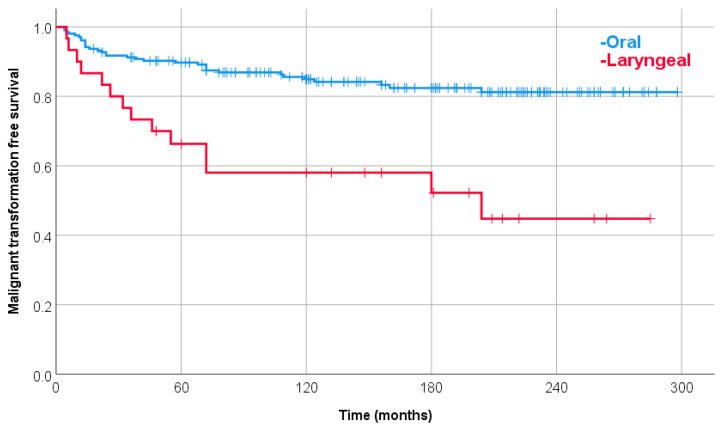
Malignant transformation-free survival with oral or laryngeal leukoplakia. The 10-year estimated malignant transformation rate was 15.1% and 42.0%, respectively (*p* < 0.0001).

The 10-year estimated malignant transformation rate of leukoplakia for all (253) patients was 18.5%. The laryngeal leukoplakia patients have a significantly increased risk of malignant transformation compared with oral patients (univariate Cox Hazard Ratio (HR): 3.13). The 10-year estimated malignant transformation rate was 42.0% and 15.1%, respectively (Figure 1).

The results of the multivariate Cox regression model, run for all patients, are shown in Table 2. The non-homogenous lesion and higher grade of dysplasia remained independent negative predictors of malignant transformation-free survival.

A separate analysis of patients with oral leukoplakia is shown in Table 3. The mean follow-up time was 149.9 months (14–328 months). Smoking, the lesion site, the lesion type, dysplasia and the grade of dysplasia had significant effects on malignant transformation-free survival. In the multivariate Cox regression model, only dysplasia and the grade of dysplasia remained independent predictors of malignant transformation-free survival (Table 4). Four patients with oropharyngeal leukoplakia developed cancer. None of them had an HPV-positive histology with a p16 immunohistochemistry examination.

The mean follow-up time for laryngeal patients was 140.9 months (36–285 months). To separately evaluate the patients with laryngeal leukoplakia, some of the subgroups have underpowered materials regarding the number of patients and malignant events (Table 5). The rate of malignant events was significantly higher only for patients with dysplastic leukoplakia: no or yes, 11.1% and 80%, respectively (*p* = 0.002). In total, 73% (11 of 15) of patients with dysplastic leukoplakia were smokers. The 10-year estimated malignant-free survival was 88.9% and 30.5%, respectively (*p* = 0.002). The presence of dysplasia significantly increased the risk of malignant transformation (HR: 12.43; 95% Confidence Interval: 1.59–97.38; *p* = 0.016). Furthermore, seven dysplastic lesions progressed to a higher grade over the follow-up time.

## 4. Discussion

In the present study, we evaluated the malignant transformation rate of patients with oral or laryngeal leukoplakia. The 10-year estimated rates were 15.1% and 42.0%, respectively (*p* < 0.0001). The mean time to malignant transformation was longer with 3 months for laryngeal leukoplakia compared to an oral lesion, but the difference was not significant (laryngeal or oral: 55.6 months and 52.7 months, respectively). The relative risk for the malignant transformation of leukoplakia was more than three times higher for patients with laryngeal cancer. The dysplasia of leukoplakia significantly increased the malignant transformation rate in both groups. The grade of dysplasia also had a significant effect on the malignant transformation (*p* < 0.0001). Up until now, no study compared the malignant transformation risk of leukoplakia of the two anatomical sites. The survival with leukoplakia-associated cancer was similar in the two groups.

We made a detailed analysis for patients with oral leukoplakia because of the appropriate number of patients (*n* = 221) with a long follow-up time (mean: 149.9 months). The 10-year estimated rate of the malignant transformation of oral leukoplakia was 15.1%. A smoking habit (never vs. ever), the lesion type (homogenous vs. non-homogenous), dysplasia (yes vs. no) and the grade of dysplasia had a significant effect on malignant transformation-free survival. The presence of dysplasia and high-grade dysplasia remained independent negative predictors of malignant transformation-free survival in a multivariate Cox model. In an earlier study from China, the mean follow-up time was 5.3 years and the malignant transformation rate was 17.9%. High-grade epithelial dysplasia proved to be an independent predictor of malignant transformation-free survival, but a smoking habit did not [16]. Two years later, also from Shanghai [17], a multivariate analysis revealed that four factors including a patient age of >60 years, a lesion located on the lateral/ventral tongue, a non-homogenous lesion and high-grade dysplasia were significant independent indicators for the malignant transformation of oral leukoplakia. Sequential biopsies were suggested for high-risk patients for the early detection of a malignant event. In a study by de Vincente et al. [18], histopathological grading was also significantly associated with oral cancer risk and was found to be a significant independent predictor in the multivariate analysis. In a review study (11,423 patients) by Warnakulasuriya and Ariyawardana, the malignant transformation rate for oral leukoplakia had a wide range between 0.13% and 34.0%. Significant determinants of the malignant transformation of oral leukoplakia included an advanced age, the female sex, leukoplakia exceeding 200 mm^2^, the non-homogeneous type and higher grades of dysplasia. They concluded that the determinants exposed in the review require further investigation [3]. In another review and meta-analysis of the last 5 years, 16,604 patients with oral leukoplakia were involved. The proportion of malignant transformation varied between 1.1% and 40.8%. The female sex, non-homogeneous clinical type and presence of epithelial dysplasia were significantly related to malignant transformation. Other risk factors previously suggested did not show significant results [19]. In a Swedish study [20] of the 234 included patients, with a median follow up of 9 years, 27 (11.5%) developed oral squamous cell carcinoma. Among the clinicopathologic factors investigated, non-homogeneous oral leukoplakia, leukoplakia with dysplasia and leukoplakia localized to the tongue showed statistically significant increased rates of malignant transformation in the multivariate Cox regression analysis. In our patients, oral leukoplakia with dysplasia or non-homogenous lesions were also independent predictors of malignant transformation, but the most common site of malignant transformation was the gingiva. The mean follow-up time of our patients was longer than 9 years—148.8 months. Malignant transformations (*n* = 6) were seen even over 10 years after the diagnosis of leukoplakia. Our long-term follow-up period could contribute to the increased rate of malignant transformation. The longest time to the malignant transformation of oral leukoplakia was observed in a Spanish study [21]—15 years and 2 months. In our patients, the longest time to the malignant transformation of leukoplakia was 204 months, which is longer with 22 months. The severe dysplasia also significantly increased the rate of malignant transformation in their patients. Furthermore, the rate of patients with early-stage cancer was much better than in our patients, 19.2% and 48.4%, respectively. The majority of the patients of Jäwert et al. [22] had 3–6 month follow-up intervals conducted by a specialist and the oral potentially malignant disorder-associated cancers were diagnosed in an early stage, which improved the survival with cancer significantly. A large cohort study from Northern California included 4886 oral leukoplakia patients with 4.62 mean years of follow up. The risk of progression to oral cancer significantly increased with the grade of dysplasia. One of their key observations was that a large proportion of oral cancer arose from leukoplakias diagnosed initially as non-dysplastic lesions. They suggested a biopsy of all clinically diagnosed leukoplakias because of the modest accuracy of the decision to biopsy leukoplakia [23]. In our cohort study, 77 patients with oral leukoplakia were not subjected to biopsy and only 3.9% of them developed oral cancer. The oral cancer rate of patients with a non-dysplastic histology was also low—5.1%. Therefore, we do not suggest routine biopsy of all leukoplakias. They need close (two times/year) monitoring for signs of early cancer.

To separately evaluate the patients with laryngeal leukoplakia, some of the subgroups have underpowered materials regarding the number of patients and malignant events. The presence of dysplasia increased the risk of malignant transformation in our patients. In a Chinese study [24], 215 patients were analyzed. The rate of no dysplastic leukoplakia was very high—54.4%. In our study, this rate was only 11.1%. The multivariate analysis showed that the pathological classification of moderate to severe dysplasia was the independent risk factor for the recurrence and malignant transformation of laryngeal leukoplakia (*p* < 0.05). In a study from Israel [25], severe dysplasia at the initial diagnosis and heavy smoking were risk factors of malignant transformation. In our patients, the malignant transformation rate was high even with grade I–II leukoplakia. However, initial dysplasia progressed over the follow-up period, and 73% of our patients with dysplasia were smokers. A study by Zhang et al. [26] included 32 patients with laryngeal leukoplakia. The malignant transformation rates for mild, moderate and severe dysplasia were 33%, 75% and 75%, respectively. Our patients with dysplasia also had high malignant transformation rates. Leduchowska et al. [27] used endoscopic (plaque morphology) and stroboscopic (mucosal wave assessment) examinations to estimate the degree of dysplasia in vocal fold leukoplakia. The rate of low- or high-grade dysplastic leukoplakia was 61.8% and 38.2%, respectively. Their findings can be used to guide a decision regarding immediate biopsy or watchful waiting. Our patients had high (75%) malignant transformation even with grade I dysplasia.

## 5. Conclusions

Patients with oral or laryngeal dysplastic leukoplakia have an increased risk of malignant transformation, but the risk is about three times higher for patients with laryngeal leukoplakia. There is no significant difference between the groups regarding survival with leukoplakia-associated cancer. Patients with non-dysplastic lesions have a low risk of malignant transformation especially in the oral group. An immediate surgical complete excision and strict and long-term follow up are suggested for high-risk patients to diagnose cancer in an early stage and to control late (over 10 years) malignant events.

## Figures and Tables

**Table 1:** Results of all patients using characteristics.

Characteristic	Malignant Events *n* (%)	*p*	10-Year MTFS % (±SD)	*p*	Univariate Cox HR (CI 95%)	*p*
All patients	64/253 (25.3%)		81.5 ± 2.6			
Gender						
female	29/138 (21%)	0.11	86.0 ± 3.2	0.061	1	0.065
male	35/115 (30.4%)		76.3 ± 4.3		1.72 (0.97–3.06)	
Age (years)						
≤60	38/152 (25%)	>0.999	79.8 ± 4.7	0.546	1	0.548
>60	26/101 (25.7%)		81.9 ± 3.3		1.20 (0.66–2.16)	
Smoking						
never	5/34 (14.7%)	0.0004	87.8 ± 5.7	0.004	1	0.009
past and present	47/96 (49%)		62.6 ± 5.6		4.01 (1.42–11.30)	
unknown	12/123 (9.8%)					
Oral vs. laryngeal						
oral	48/221 (21.7%)	0.002	84.9 ± 2.6	<0.0001	1	<0.0001
laryngeal	16/32 (50%)		58.0 ± 9.4		3.13 (1.71–5.72)	
Lesion type						
homogenous	53/236 (22.5%)	0.0004	83.2 ± 2.6	0.003	1	0.005
non-homogenous	11/17 (64.7%)		53.8 ± 13.8		3.46 (1.46–8.18)	
Biopsy						
no	4/83 (4.8%)	<0.0001	95.9 ± 2.4	<0.0001	1	<0.0001
yes	60/170 (35.3%)		74.0 ± 3.7		6.48 (2.33–18.05)	
Histology						
no dysplasia	5/88 (5.7%)	<0.0001	95.3 ± 2.3	<0.0001	1	<0.0001
grade I dysplasia	7/29 (24.1%)		79.3 ± 7.5		4.76 (1.51–15.03)	
grade II dysplasia	16/20 (80%)		18.3 ± 10.3		32.62 (11.51–92.44)	
grade III dysplasia	15/16 (93.8%)		18.8 ± 9.8		29.79 (10.73–82.73)	
in situ cancer	5					
invasive cancer	12					
Dysplasia						
no	5/88 (5.7%)	<0.0001	95.3 ± 2.3	<0.0001	1	<0.0001
yes	38/65 (58.5)		45.9 ± 6.4		14.58 (5.71–37.24)	
Dysplasia						
low grade (I)	7/29 (24.1%)	<0.0001	79.3 ± 7.5	<0.0001	1	<0.0001
high grade (II, III)	31/36 (86.1%)		18.3 ± 7.0		6.78 (2.94–15.63)	

MTFS, malignant transformation-free survival; SD, standard deviation; HR, hazard ratio; CI, confidence interval.

**Table 2:** Multivariate model for all patients.

Characteristic	Multivariate Cox HR (CI 95%)	*p*
Smoking		
never	1	0.142
past and present	2.49 (0.74–8.44)	
Lesion type		
homogenous	1	0.014
non-homogenous	3.74 (1.31–10.09)	
Dysplasia		
no	1	<0.0001
low grade (I)	4.23 (1.11–16.16)	
high grade (II, III)	12.25 (4.10–36.66)	

HR, hazard ratio; CI, confidence interval.

**Table 3:** Results of patients with oral leukoplakia using characteristics.

Characteristic	Malignant Events *n* (%)	*p*	10-Year MTFS %, SD	*p*	Univariate Cox HR (CI 95%)	*p*
All patients	48/221 (21.7%)		84.9 ± 2.6			
Gender						
female	26/128 (20.3%)	0.621	85.8 ± 3.3	0.756	1	0.756
male	22/93 (23.7%)		83.9 ± 4.1		1.12 (0.56–2.23)	
Age (years)						
≤60	25/126 (19.8%)	0.51	86.2 ± 3.2	0.235	1	0.239
>60	23/95 (24.2%)		81.9 ± 4.8		1.52 (0.76–3.02)	
Smoking						
never	4/33 (12.1%)	0.0004	90.6 ± 5.2	0.004	1	0.009
past and present	35/73 (48%)		63.4 ± 6.5		4.93 (1.48–16.38)	
unknown	9/115 (7.8%)					
Lesion site *						
tongue	14/60 (23.3%)	0.003	82.6 ± 5.4	0.007	2.89 (0.90–9.20)	0.003
sublingual	6/24 (25%)		76.7 ± 9.2		3.67 (0.98–13.66)	
palate	3/8 (37.5%)		71.4 ± 17.1		5.00 (0.92–27.35)	
buccal	6/60 (10%)		96.5 ± 2.4		1	
gingiva	6/13 (46.1%)		52.4 ± 15.7		7.17 (1.93–26.74)	
lips	0/27 (0%)		100		0	
oropharyngeal	4/10 (40%)		75.0 ± 15.3		4.24 (0.78–23.13)	
multifocal	9/19 (47.4%)		73.3 ± 11.4		5.47 (1.47–20.39)	
Lesion type						
homogenous	37/203 (18.2%)	0.0002	87.0 ± 2.6	<0.0001	1	0.001
non-homogenous	11/18 (61.1%)		53.8 ± 13.8		4.57 (1.88–11.13)	
Biopsy						
no	3/77 (3.9%)	<0.0001	96.9 ± 2.2	<0.0001	1	<0.0001
yes	45/144 (31.3%)		77.9 ± 3.8		6.60 (2.01–21.63)	
Histology						
no dysplasia	4/79 (5.1%)	<0.0001	96.1 ± 2.2	<0.0001	1	<0.0001
grade I dysplasia	4/25 (16%)		88.0 ± 6.5		3.43 (0.86–13.75)	
grade II dysplasia	12/15 (80%)		22.2 ± 12.2		40.71 (11.91–139.11)	
grade III dysplasia	10/10 (100%)		0		54.33 (15.15–194.84)	
in situ cancer	4					
invasive cancer	11					
Dysplasia						
no	4/79 (5.1%)	<0.0001	96.1 ± 2.2	<0.0001	1	<0.0001
yes	26/50 (52%)		50.0 ± 7.4		13.79 (4.79–39.72)	
Dysplasia						
low grade (I)	4/25 (16%)	<0.0001	88.0 ± 6.5	<0.0001	1	<0.0001
high grade (II, III)	22/25 (88%)		11.3 ± 7.1		14.63 (4.28–50.03)	

MTFS, malignant transformation-free survival; SD, standard deviation; HR, hazard ratio; CI, confidence interval; * p16 immunohistochemistry was negative for oropharyngeal patients.

**Table 4:** Multivariate Cox model for patients with oral leukoplakia.

Characteristic	Multivariate Cox HR (CI 95%)	*p*
Dysplasia		
no	1	<0.0001
low grade (I)	2.47 (0.41–15.03)	
high grade (II, III)	18.19 (4.71–70.25)	
Lesion type		
homogenous	1	0.063
non-homogenous	2.79 (0.95–8.24)	
Smoking		
never	1	0.098
past and present	3.51 (0.79–15.51)	

HR, hazard ratio; CI, confidence interval.

**Table 5:** Malignant events of patients with laryngeal leukoplakia using characteristics.

Characteristic	Malignant Events *n* (%)	*p*
All patients	16/32 (50%)	
Gender		
female	3/10 (30%)	0.252
male	13/22 (59.1%)	
Age (years)		
≤60	13/26 (50%)	>0.999
>60	3/6 (50%)	
Smoking		
never	1/1 (100%)	>0.999
past and present	12/23 (52.2%)	
unknown	3/8 (37.5%)	
Site		
unilateral	9/20 (45%)	0.717
bilateral	7/12 (58.3%)	
Lesion type		
homogenous	16/32 (50%)	>0.999
non-homogenous	0/0	
Biopsy		
no	1/6 (16.7%)	0.172
yes	15/26 (57.7%)	
Histology		
in situ cancer	1	
invasive cancer	1	
Dysplasia		
no	1/9 (11.1%)	0.002
yes	12/15 (80%)	
Dysplasia		
low grade (I)	3/4 (75%)	>0.999
high grade (II, III)	9/11 (81.8%)	

## Data Availability

The data can be found in the database of the National Institute of Oncology.

## References

[1] Baran C.A., Agaimy A., Wehrhan F., Weber M., Hille V., Brunner K., Wickenhauser C., Siebolts U., Nkenke E., Kesting M. (2019). MAGE-A expression in oral and laryngeal leukoplakia predicts malignant transformation. Mod. Pathol..

[2] van Hulst A.M., Kroon W., van der Linden E.S., Nagtzaam L., Ottenhof S.R., Wegner I., Gunning A.C., Grolman W., Braunius W. (2016). Grade of dysplasia and malignant transformation in adults with premalignant laryngeal lesions. Head Neck.

[3] Warnakulasuriya S., Ariyawardana A. (2016). Malignant transformation of oral leukoplakia: A systematic review of observational studies. J. Oral Pathol. Med..

[4] Weller M., Nankivell P., McConkey C., Paleri V., Mehanna H. (2010). The risk and interval to malignancy of patients with laryngeal dysplasia; a systematic review of case series and meta-analysis. Clin. Otolaryngol..

[5] Isenberg J.S., Crozier D.L., Dailey S.H. (2008). Institutional and Comprehensive Review of Laryngeal Leukoplakia. Ann. Otol. Rhinol. Laryngol..

[6] Iocca O., Sollecito T.P., Alawi F., Weinstein G.S., Newman J.G., De Virgilio A., Di Maio P., Spriano G., López S.P., Shanti R.M. (2020). Potentially malignant disorders of the oral cavity and oral dysplasia: A systematic review and meta-analysis of malignant transformation rate by subtype. Head Neck.

[7] Kuribayashi Y., Tsushima F., Morita K.-I., Matsumoto K., Sakurai J., Uesugi A., Sato K., Oda S., Sakamoto K., Harada H. (2015). Long-term outcome of non-surgical treatment in patients with oral leukoplakia. Oral Oncol..

[8] Warnakulasuriya S., Kujan O., Aguirre-Urizar J.M., Bagan J.V., González-Moles M., Kerr A.R., Lodi G., Mello F.W., Monteiro L., Ogden G.R. (2021). Oral potentially malignant disorders: A consensus report from an international seminar on nomenclature and classification, convened by the WHO Collaborating Centre for Oral Cancer. Oral Dis..

[9] Hsue S.S., Wang W.C., Chen C.H., Lin C.C., Chen Y.K., Lin L.M. (2007). Malignant transformation in 1458 patients with potentially ma-lignant oral mucosal disorders: A follow-up study based in a Taiwanese hospital. J. Oral Pathol. Med..

[10] van der Waal I. (2009). Potentially malignant disorders of the oral and oropharyngeal mucosa; terminology, classification and present concepts of management. Oral Oncol..

[11] Karatayli-Ozgursoy S., Pacheco-Lopez P., Hillel A.T., Best S.R., Bishop J.A., Akst L.M. (2015). Laryngeal dysplasia, demographics, and treatment: A single-institution, 20-year review. JAMA Otolaryngol. Head Neck Surg..

[12] Eversole L.R. (2009). Dysplasia of the upper aerodigestive tract squamous epithelium. Head Neck Pathol..

[13] Gale N., Zidar N., Poljak M., Cardesa A. (2014). Current views and perspectives on classification of squamous intraepithelial lesions of the head and neck. Head Neck Pathol..

[14] Kaplan E., Meier P. (1958). Nonparametric estimation from incomplete observations. J. Am. Stat. Assoc..

[15] Cox D.R. (1972). Regression models and life-tables. J. R. Stat. Soc..

[16] Liu W., Wang Y.-F., Zhou H.-W., Shi P., Zhou Z.-T., Tang G.-Y. (2010). Malignant transformation of oral leukoplakia: A retrospective cohort study of 218 Chinese patients. BMC Cancer.

[17] Liu W., Shi L.J., Wu L., Feng J.Q., Yang X., Li J., Zhou Z.T., Zhang C.P. (2012). Oral cancer development in patients with leukoplakia—Clinicopathological factors affecting outcome. PLoS ONE.

[18] de Vicente J.C., del Molino P.D.-P., Rodrigo J.P., Allonca E., Hermida-Prado F., Granda-Díaz R., Santamarta T.R., García-Pedrero J.M. (2019). SOX2 Expression Is an Independent Predictor of Oral Cancer Progression. J. Clin. Med..

[19] Aguirre-Urizar J.M., de Mendoza I.L.I., Saman Warnakulasuriya S. (2021). Malignant transformation of oral leukoplakia: Systematic review and meta-analysis of the last 5 years. Oral Dis..

[20] Jäwert F., Pettersson H., Jagefeldt E., Holmberg E., Kjeller G., Öhman J. (2021). Clinicopathologic factors associated with malignant transformation of oral leukoplakias: A retrospective cohort study. Int. J. Oral Maxillofac. Surg..

[21] Bagan J., Martorell M., Cebrián J.L., Rubert A., Bagán L., Mezquida C., Hervás D. (2022). Effect of clinical and histologic features on time to malignancy in 224 cases of oral leukoplakia treated by surgery. Clin. Oral Investig..

[22] Jäwert F., Nyman J., Olsson E., Adok C., Helmersson M., Öhman J. (2021). Regular clinical follow-up of oral potencially malignant dis-orders results in improved survival for patients who develop oral cancer. Oral Oncol..

[23] Chaturvedi A.K., Udaltsova N., Engels E.A., Katzel J.A., Yanik E.L., Katki H.A., Lingen M.W., Silverberg M.J. (2020). Oral Leukoplakia and Risk of Progression to Oral Cancer: A Population-Based Cohort Study. Gynecol. Oncol..

[24] Niu Y.Y., Wang J., Huo H., Jin X.F., Li W.Y., Gao Z.Q. (2018). Clinical analyses of 263 patients with laryngeal leukoplakia. Zhonghua Er Bi Yan Hou Tou Jing Wai Ke Za Zhi.

[25] Jabarin B., Pitaro J., Marom T., Muallem-Kalmovich L. (2018). Dysplastic Changes in Patients with Recurrent Laryngeal Leukoplakia: Importance of Long-Term Follow-Up. Isr. Med. Assoc. J..

[26] Zhang H., Chen X.M., Li Z.H. (2000). Clinical analysis of vocal cord leukoplakia in 32 cases. Lin Chuang Er Bi Yan Hou Ke Za Zhi J. Clin. Otorhinolaryngol..

[27] Leduchowska A., Morawska J., Pietruszewska W. (2022). Videolaryngoendoscopic and Stroboscopic Evaluation in Predicting the Ma-lignancy Risk of Vocal Fold Leukoplakia. J. Clin. Med..

